# CT psoas calculations on the prognosis prediction of emergency laparotomy: a single-center, retrospective cohort study in eastern Asian population

**DOI:** 10.1186/s13017-022-00435-x

**Published:** 2022-06-03

**Authors:** Xiao-Lin Wu, Jie Shen, Ci-Dian Danzeng, Xiang-Shang Xu, Zhi-Xin Cao, Wei Jiang

**Affiliations:** Department of Gastrointestinal Surgery, Tongji Hospital of Tongji Medical College, Huazhong University of Science and Technology, 1095 Jiefang Av., Wuhan, 430030 Hubei People’s Republic of China

**Keywords:** Emergency laparotomy, Sarcopenia, Psoas major

## Abstract

**Background:**

Emergency laparotomy (EL) has a high mortality rate. Clinically, frail patients have a poor tolerance for EL. In recent years, sarcopenia has been used as an important indicator of frailty and has received much attention. There have been five different calculation methods of psoas for computed tomography (CT) to measure sarcopenia, but lack of assessment of these calculation methods in Eastern Asian EL patients.

**Methods:**

We conducted a 2-year retrospective cohort study of patients over 18 years of age who underwent EL in our institution. Five CT measurement values (PMI: psoas muscle index, PML3: psoas muscle to L3 vertebral body ratio, PMD: psoas muscle density, TPG: total psoas gauge, PBSA: psoas muscle to body face area ratio) were calculated to define sarcopenia. Patients with sarcopenia defined by the sex-specific lowest quartile of each measurement were compared with the rest of the cohort. The primary outcome was "ideal outcome", defined as: (1) No postoperative complications of Clavien-Dindo Grade ≥ 4; (2) No mortality within 30 days; (3) When discharged, no need for fluid resuscitation and assisted ventilation, semi-liquid diet tolerated, and able to mobilize independently. The second outcome was mortality at 30-days. Multivariate logistic regression and receiver operating characteristic (ROC) analysis were used.

**Results:**

Two hundred and twenty-eight patients underwent EL met the inclusion criteria, 192 (84.2%) patients had an ideal outcome after surgery; 32 (14%) patients died within 30 days. Multivariate analysis showed that, except PMD, each calculation method of psoas was independently related to clinical outcome (ideal outcome: PML3, *P* < 0.001; PMI, *P* = 0.001; PMD, *P* = 0.157; TPG, *P* = 0.006; PBSA, *P* < 0.001; mortality at 30-days: PML3, *P* < 0.001; PMI, *P* = 0.002; PMD, *P* = 0.088; TPG, *P* = 0.002; PBSA, *P* = 0.001). In ROC analysis, the prediction model containing PML3 had the largest area under the curve (AUC) value (AUC value = 0.922 and 0.920, respectively).

**Conclusion:**

The sarcopenia determined by CT psoas measurements is significantly related to the clinical outcome of EL. The calculation of CT psoas measurement is suitable for application in outcome prediction of EL. In the future, it is necessary to develop a scoring tool that includes sarcopenia to evaluate the risk of EL better.

**Supplementary Information:**

The online version contains supplementary material available at 10.1186/s13017-022-00435-x.

## Introduction

Emergency laparotomy has a high mortality rate [1], and decision-making for surgical treatment is a challenge for surgeons [2]. Accurate risk prediction for patients is critical for optimizing surgical treatment decisions and allocation of medical resources [3]. In the past, the risk prediction of emergency laparotomy generally lacked the inclusion of the parameter of "frailty"[3].

Sarcopenia was first described by Irwin H. Rosenberg in 1988 and used to describe the age-related loss of skeletal muscle quantity and quality [4]. European Working Group on Sarcopenia in Older People (EWGSOP) clarified the definition of sarcopenia in 2010: sarcopenia is a syndrome characterized by progressive and comprehensive loss of skeletal muscle mass and muscle strength, accompanied by the risk of adverse consequences, such as physical disability, poor quality of life, and death [5]. In 2018, EWGSOP updated the consensus on sarcopenia and encouraged research in this field [6]. The role of the quality and quantity of skeletal muscle in clinical outcomes has received increasing attention.

The quantity and quality of muscles should be based on computed tomography (CT) or magnetic resonance imaging (MRI) as the gold standard [6–9]. In practical applications, imaging can be used as a routine examination item for diagnosis to evaluate the state of skeletal muscles. In recent studies, it was common to use CT muscle measurements to define sarcopenia. The relevant measurement was selected at the L3 level, where the level of skeletal muscle can well reflect the level of the whole body [10, 11].

Many studies suggested that sarcopenia was associated with a poor prognosis of emergency laparotomy [2, 12–17]; however, related research was mainly conducted in medical centers in western developed countries. It is still unclear whether the same conclusion is suitable for people in developing countries in East Asia. Due to differences in lifestyle and cultural background, there is a certain degree of body composition difference between the two groups of people [18].

In the reported studies, there were five different psoas muscle calculation methods. Researchers had compared the prediction of three of them, psoas muscle index (PMI), psoas muscle to L3 vertebral body ratio (PML3) and psoas muscle to body face area ratio (PBSA), in European populations [12]. Lu et al. defined total psoas gauge (TPG) in the prognosis study of gastric cancer, proved it was an independent risk factor in the prediction model of postoperative complications [19]. In addition, studies showed that psoas muscle density (PMD) was associated with the prognosis of emergency laparotomy [2, 16]. No studies have compared the predictive capability of all these psoas major muscle calculations on prognosis yet.

In this study, we aimed to verify the universality of the conclusion that sarcopenia affected the prognosis of emergency laparotomy in a different population setting. In addition, we compared the ability of five different psoas calculations to predict clinical outcomes, which could be the basis for developing a more reliable risk prediction model.

## Methods

### Hospital

Our institution is a tertiary medical center located in central China. It has a case database, and medical records can be browsed in the local area network. The hospital's institutional review board passed the ethical approval of the study.

### Patients

This study selected adult patients who underwent emergency laparotomy in our prospective database from September 1, 2019, to August 31, 2021. All patients' information was retrieved from our hospital's database, including demographics, comorbidities, preoperative laboratory inspection results, weight, height, body mass index (BMI), American Society of Anesthesiologists (ASA) score, surgical procedures, intraoperative conditions, and prognosis, etc. The Charlson Comorbidity Index (CCI) [20] was calculated according to the retrieved data. The sepsis diagnostic criterion was referred to Sepsis-3 [21].

### Inclusion and exclusion criteria

Inclusion criteria: (1) Age greater than 18 years; (2) Emergency laparotomy in our hospital; (3) CT scan before operation.

Exclusion criteria: (1) Under 18 years of age; (2) Elective surgery or non-open surgery; (3) Emergency laparotomy for patients with severe trauma; (4) Loss of relevant data; (5) The preoperative CT scan is a contrast-enhanced CT, poor CT quality, or the patient with blood vessels stents, ureteral stents, artificial joints, or other implants.

**Fig. 1:**
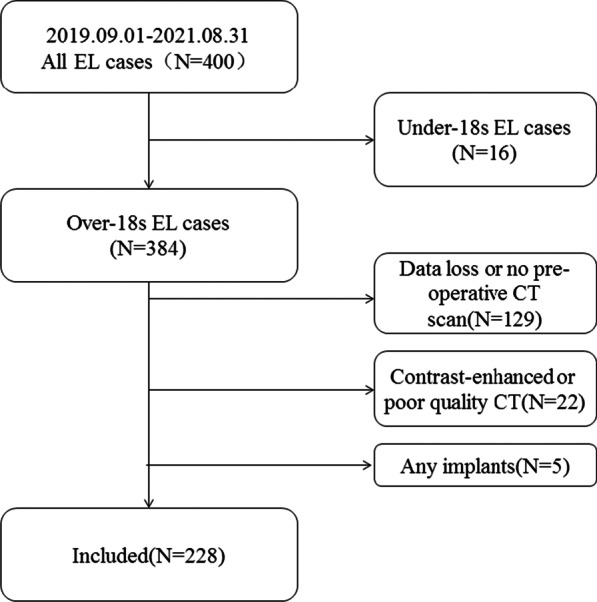
Screening flowchart

The patient selection flowchart is shown in Fig. 1.

### Surgery

Surgical procedures were dichotomized into minor surgery (perforation repairment, appendectomy, adhesiolysis, exploratory, abdominal hernia, reduction of volvulus, drainage of abscess) and major surgery (small bowel resection, colon colostomy, right colectomy, left colectomy, other colorectal resection, Hartmann's, removal of foreign body, other tumor resection, gastrectomy, enterostomy, resection of Meckel's diverticulum). In the case of multiple procedures in a single surgery, we made statistics based on the higher-grade procedure. For example, when both "small bowel resection" and "appendectomy" were performed in a single surgery, we would record the procedure as "small bowel resection" rather than "appendectomy".

### Imaging data

**Fig. 2:**
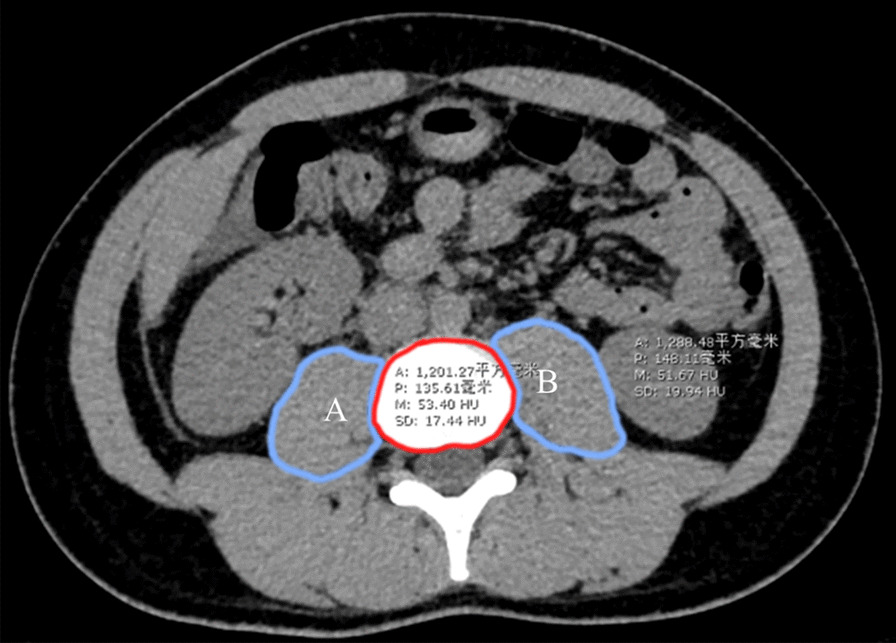
Example of measuring left psoas area (LPA), the right psoas area (RPA), the left psoas muscle density (LPMD), the right psoas muscle density (RPMD), and the L3 vertebral body area at the inferior end-plate level of the L3 vertebral body (The blue outline shows psoas, A for the right psoas and B for the left psoas; the red outline shows L3 vertebral body). The five psoas calculations were then calculated according to the following equations: PMI (mm^2^/m^2^) = TPA/height (m)^2^, PML3 = TPA/area of L3 vertebral body, PMD (HU) = (LPA × LPMD + RPA × RPMD)/TPA, TPG (AU) = PMI × PMD, PBSA (mm^2^/m^2^) = TPA/(height (cm) × weight (kg)/3600)½. (TPA = LPA + RPA). L3 for third lumbar vertebra. PMI for psoas muscle index. TPA for total psoas area. PML3 for psoas muscle to L3 vertebral body ratio. PMD for psoas muscle density. TPG for total psoas gauge. PBSA for psoas muscle to body face area ratio

The collection and analysis of image data were conducted with Synapse workstation (version 3.2.1, Fujifilm medical systems, USA). Referred to the method previously verified by Simpson et al., we chose to collect data at the level of the inferior end-plate of the L3 (the third lumbar) vertebra [12, 13, 17], as shown in Fig. 2. The selected level should show an independent lumbar vertebral body area. The selection tool of the imaging workstation could be used to draw the area of interest (ROI), and the system would automatically generate the average CT value (HU) and area (mm^2^) of the ROI. Using this method, we measured and obtained the left psoas area (LPA), the right psoas area (RPA), the left psoas muscle density (LPMD), the right psoas muscle density (RPMD), and the L3 vertebral body area. These measurements were then used to calculate five psoas calculations below:

$$\begin{aligned} & {\text{PMI}}\,({\text{mm}}^{2} /{\text{m}}^{2} ) = {\text{TPA}}/{\text{height}}\,({\text{m}})^{2} , \\ & {\text{PML}}3 = {\text{TPA}}/{\text{area}}\,{\text{of}}\,{\text{L3}}\,{\text{vertebral}}\,{\text{body}}, \\ & {\text{PMD}}\,({\text{HU}}) = ({\text{LPA}} \times {\text{LPMD}} + {\text{RPA}} \times {\text{RPMD}})/{\text{TPA}}, \\ & {\text{TPG}}\,({\text{AU}}) = {\text{PMI}} \times {\text{PMD}}, \\ & {\text{PBSA}}\,({\text{mm}}^{2} /{\text{m}}^{2} ) = {\text{TPA}}/({\text{height}}\,({\text{cm}}) \times {\text{weight}}({\text{kg}})/3600){\raise0.7ex\hbox{$1$} \!\mathord{\left/ {\vphantom {1 2}}\right.\kern-\nulldelimiterspace} \!\lower0.7ex\hbox{$2$}}. \\ \end{aligned}$$

(TPA (total psoas area) = LPA + RPA. Body surface area (BSA) was calculated by the Mosteller formula: BSA (m^2^) = (height (cm) × weight (kg)/3600)½).

Two trained researchers completed the data collection and calculation together without knowing the patients' demographic information and prognostic information. Before collecting image information, the two researchers assessed the quality of each image in a consistent protocol to decide whether to exclude the corresponding patient. Poor image quality may affect subsequent image data analysis.

### Statistical analysis

In this study, the primary outcome parameter was the "ideal outcome", defined as: (1) No postoperative complications of Clavien-Dindo Grade ≥ 4; (2) No mortality within 30 days; (3) When discharged, no need for fluid resuscitation and assisted ventilation, semi-liquid diet tolerated, and able to mobilize independently. The second outcome was mortality at 30-days.

Regarding the previously reported method [19, 22], we obtained the lowest quartile of sex-specific psoas measurements as the cut-off value to define whether there was sarcopenia.

Normality of data distribution was determined by the Kolmogorov–Smirnov test. Normally distributed data were expressed as mean (± SD) and non-normally distributed data were expressed as median [IQR]; categorical variables were expressed as *n* (%). The analysis of continuous variables used the Mann–Whitney U test or T-test. Categorical variables used the chi-square test. Binary logistic regression was used for multivariate analysis. The receiver operating characteristic (ROC) curve was used to evaluate the model's predictive ability. The area under curve (AUC) values of models were compared using pairwise DeLong test [23]. *P* value < 0.05 was considered statistically significant. All statistical analysis was conducted in SPSS Statistics for Windows v26.0 (Armonk, NY: IBM Corp).

## Result

### Patient baseline characteristics

A total of 228 patients were enrolled in this study, including 138 (60.5%) men and 90 (39.5%) women. The average age of the population was 57.7 (± 15.8) years, and the average BMI value was 21.7 (± 3.5) kg/m^2^. Among the study population, 89 people (39.0%) had previous abdominal surgery; forty-four people (19.3%) were diagnosed with malignant tumors before or after surgery. The median Charlson Comorbidity Index score was 1.0 [0.0, 2.0]. Population baseline characteristics are shown in Table 1.

**Table 1:** Baseline characteristics

Variables	*n* (%)/mean (± SD)/median [IQR]
*Sex, n (%)*	
Male	138 (60.5%)
Female	90 (39.5%)
Age, years, mean (± SD)	57.7(± 15.8)
BMI, kg/m^2^, mean (± SD)	21.7(± 3.5)
Previous Abdominal Surgery, *n* (%)	89 (39.0%)
Charlson Comorbidities Index, median [IQR]	1.0 [0.0,2.0]
Malignancy, *n* (%)	44 (19.3%)
Sepsis, *n* (%)	99 (43.4)
Peritoneal Soiling, *n* (%)	130 (57.0%)
*ASA Score, n (%)*	
I	7 (3.1%)
II	98 (43.0%)
III	96 (42.1%)
IV	26 (11.4%)
V	1 (0.4%)

*SD* standard deviation, *IQR* interquartile range, *BMI* body mass index, *ASA* American Society of Anesthesiologists

One hundred and eighteen (51.8%) patients received major surgeries and 110 (48.2%) patients received minor surgeries. The commonest surgeries were small bowel resection (24.6%), perforation repair (18.4%), and appendectomy (11.8%). Thirty-three (14.5%) patients underwent more than one of the procedures, with the commonest surgery being small bowel resection combined with abdominal wall hernia repairment (3.5%). The operative procedures are shown in Table 2.

**Table 2:** Operative procedures

	Frequency (%)
*Major*	118 (51.8)
Small bowel resection	56 (24.6)
Colon colostomy	10 (4.4)
Right colectomy	10 (4.4)
Other colorectal resection	10 (4.4)
Hartmann's	6 (2.6)
Removal of foreign body	6 (2.6)
Other tumor resection	5 (2.2)
Gastrectomy	5 (2.2)
Enterostomy	5 (2.2)
Left colectomy	3 (1.3)
Resection of Meckel's diverticulum	2 (0.9)
*Minor*	110 (48.2)
Perforation repairment	42 (18.4)
Appendectomy	27 (11.8)
Adhesiolysis	15 (6.6)
Exploratory	11 (4.8)
Abdominal hernia	7 (3.1)
Reduction of volvulus	5 (2.2)
Drainage of abscess	3 (1.3)

### Cut-off values of psoas muscle measurement

The sex-specific cut-off values for the five psoas muscle measurements are shown in Table 3. Sarcopenia was defined as having a measurement below the sex-specific cut-off value in the cohort.

**Table 3:** Cut-off values of each psoas calculation

	PML3	PMI (mm^2^/m^2^)	PMD (HU)	TPG (AU)	PBSA (mm^2^/m^2^)
Female	0.60	375.2	27.8	106.7	633.0
Male	0.74	463.9	34.9	169.7	806.1

*PML3* psoas muscle to L3 vertebral body ratio, *PMI* psoas muscle index, *PMD* psoas muscle density, *TPG* total psoas gauge, *PBSA* psoas muscle to body face area ratio

### Sarcopenia and clinical outcome

We divided the patients into "Sarcopenia" group and "Non-Sarcopenia" group according to each of these five different calculations, respectively.

In the baseline characteristics (Table 4), except for the PMI (*P* value = 0.063), the sarcopenia defined by the psoas major measurement values was age-related. In addition, the level of serum albumin was related to the sarcopenia determined by each calculation method.

**Table 4:** Patients’ baseline characteristics: Sarcopenia versus Non-Sarcopenia

Baseline characteristic	PML3			PMI (mm^2^/m^2^)			PMD (HU/mm^2^)		
	Sarcopenia	Non-Sarcopenia	*P* value	Sarcopenia	Non-Sarcopenia	*P* value	Sarcopenia	Non-Sarcopenia	*P* value
Age, years (SD)	67.5 (11.4)	54.6 (15.7)	< 0.001	61.1 (15.2)	56.6 (15.8)	0.063	68.0 (10.4)	54.4 (15.8)	< 0.001
Male gender, *n* (%)	34 (60.7)	104 (60.5)	0.974	34 (60.7)	104 (60.5)	0.974	34 (60.7)	104 (60.5)	0.974
BMI, kg/m^2^ (SD)	20.8 (3.7)	22.0 (3.4)	0.033	20.0 (3.4)	22.2 (3.4)	< 0.001	21.3 (3.4)	21.8 (3.6)	0.368
*ASA score, n (%)*			0.028			0.030			0.170
I	0 (0.0)	7 (4.1)		0 (0.0)	7 (4.1)		0 (0.0)	7 (4.1)	
II	17 (30.4)	81 (47.1)		19 (33.9)	79 (45.9)		19 (33.9)	79 (45.9)	
III	28 (50.0)	68 (39.5)		25 (44.6)	71 (41.3)		30 (53.6)	66 (38.4)	
IV	11 (19.6)	15 (8.7)		12 (21.4)	14 (8.1)		7 (12.5)	19 (11.0)	
V	0 (0.0)	1 (0.6)		0 (0.0)	1 (0.6)		0 (0.0)	1 (0.6)	
Charlson Comorbidities Index, median [IQR]	1.0 [0.0,2.0]	1.0 [0.0,2.0]	0.484	1.0 [0.0,2.0]	1.0 [0.0,2.0]	0.860	1.0 [0.0,2.0]	1.0 [0.0,2.0]	0.111
Previous abdominal surgery, *n* (%)	20 (35.7)	69 (40.1)	0.558	25 (44.6)	64 (37.2)	0.322	22 (39.3)	67 (39.0)	0.965
Sepsis, *n* (%)	30 (53.6)	69 (40.1)	0.078	30 (53.6)	69 (40.1)	0.078	31 (55.4)	68 (39.5)	0.038
Malignancy, *n* (%)	15 (26.8)	29 (16.9)	0.102	20 (35.7)	24 (14.0)	< 0.001	11 (19.6)	33 (19.2)	0.940
ALB, g/L (SD)	34.3 (5.9)	37.8 (6.8)	0.001	33.5 (5.9)	38.1 (6.7)	< 0.001	34.0 (5.9)	37.9 (6.8)	< 0.001
Hb, g/L (SD)	117.7 (26.9)	122.7 (28.6)	0.248	117.1 (27.6)	122.9 (28.3)	0.178	118.0 (23.4)	122.7 (30.0)	0.280

Baseline characteristic	TPG (AU)			PBSA (mm^2^/m^2^)		
	Sarcopenia	Non-Sarcopenia	*P* value	Sarcopenia	Non-Sarcopenia	*P* value
Age, years (SD)	65.8 (12.6)	55.1 (15.8)	< 0.001	63.1 (14.0)	56.0 (15.9)	0.003
Male gender, *n* (%)	34 (60.7)	104 (60.5)	0.974	34 (60.7)	104 (60.5)	0.974
BMI, kg/m^2^ (SD)	20.4 (3.1)	22.1 (3.5)	0.002	21.2 (3.6)	21.8 (3.5)	0.220
*ASA score, n (%)*			0.090			0.003
I	0 (0.0)	7 (4.1)		0 (0.0)	7 (4.1)	
II	20 (35.7)	78 (45.3)		16 (28.6)	82 (47.7)	
III	25 (44.6)	71 (41.3)		27 (48.2)	69 (40.1)	
IV	11 (19.6)	15 (8.7)		13 (23.2)	13 (7.6)	
V	0 (0.0)	1 (0.6)		0 (0.0)	1 (0.6)	
Charlson Comorbidities Index, median [IQR]	1.0 [0.0,2.0]	1.0 [0.0,2.0]	0.353	1.0 [0.0,2.0]	1.0 [0.0,2.0]	0.257
Previous Abdominal Surgery, *n* (%)	23 (41.1)	66 (38.4)	0.719	26 (46.4)	63 (36.6)	0.192
Sepsis, *n* (%)	31 (55.4)	68 (39.5)	0.038	33 (58.9)	66 (38.4)	0.007
Malignancy, *n* (%)	15 (26.8)	29 (16.9)	0.102	35.7	14.0	< 0.001
ALB, g/L (SD)	33.8 (5.4)	38.0 (6.9)	< 0.001	33.8 (6.02)	38.0 (6.7)	< 0.001
Hb, g/L (SD)	116.6 (27.2)	123.1 (28.4)	0.136	119.0 (26.8)	122.3 (28.6)	0.442

*PML3* psoas muscle to L3 vertebral body ratio, *PMI* psoas muscle index, *PMD* psoas muscle density, *TPG* total psoas gauge, *PBSA* psoas muscle to body face area ratio, *BMI* body mass index, *ASA* American Society of Anesthesiologists, *IQR* interquartile range, *ALB* albumin, *HB* hemoglobin

*P* value < 0.05 was considered statistically significant

Regarding surgical outcome (Table 5), sarcopenia was associated with the occurrence of complications with Clavien-Dindo grade ≥ 2. Except PMD (*P* value = 0.115) and TPG (*P* value = 0.115), sarcopenia was also associated with complications with Clavien-Dindo grade ≥ 3. Besides, the sarcopenia defined by each psoas major measurement value was related to respiratory infection; only the sarcopenia defined by PMD (*P* value = 0.036) was related to abdominal infection.

**Table 5:** Patients’ surgical outcomes: Sarcopenia versus Non-Sarcopenia

Surgical outcome	PML3			PMI (mm^2^/m^2^)			PMD (HU/mm^2^)		
	Sarcopenia	Non-Sarcopenia	*P* value	Sarcopenia	Non-Sarcopenia	*P* value	Sarcopenia	Non-Sarcopenia	*P* value
*Complication*									
CD grade ≥ 2, *n* (%)	33 (58.9)	56 (32.6)	< 0.001	89 (53.6)	59 (34.3)	0.010	29 (51.8)	60 (34.9)	0.024
CD grade ≥ 3, *n* (%)	13 (23.2)	21 (12.2)	0.045	14 (25.0)	20 (11.6)	0.015	12 (21.4)	22 (12.8)	0.115
Respiratory infection, *n* (%)	21 (37.5)	29 (16.9)	0.001	18 (32.1)	32 (18.6)	0.033	20 (35.7)	30 (17.4)	0.004
Abdominal infection, *n* (%)	10 (17.9)	30 (17.4)	0.943	9 (16.1)	31 (18.0)	0.739	15 (26.8)	25 (14.5)	0.036
Wound infection, *n* (%)	4 (7.1)	9 (5.2)	0.592	5 (8.9)	8 (4.7)	0.231	4 (7.1)	9 (5.2)	0.592
Leakage, *n* (%)	5 (8.9)	7 (4.1)	0.157	4 (7.1)	8 (4.7)	0.468	4 (7.1)	8 (4.7)	0.468
Ideal Outcome, *n* (%)	35 (62.5)	157 (91.3)	< 0.001	37 (66.1)	155 (90.1)	< 0.001	40 (71.4)	152 (88.4)	0.003
Mortality at 30-days, *n* (%)	19 (33.9)	13 (7.6)	< 0.001	17 (30.4)	15 (8.7)	< 0.001	15 (26.8)	17 (9.9)	0.002
Mortality at hospital, *n* (%)	0 (0.0)	4 (2.3)	0.250	1 (1.8)	3 (1.7)	0.984	0 (0.0)	4 (2.3)	0.250
Readmission with 30-days, *n* (%)	1 (1.8)	7 (4.1)	0.420	2 (3.6)	6 (3.5)	0.977	3 (5.4)	5 (2.9)	0.387
Length of stay, d, median [IQR]	9 [6.25,14.75]	9 [7, 12]	0.793	8.5 [6,13.75]	9 [7, 12]	0.542	9.5 [7,14.5]	9 [7, 12]	0.475
ICU Stay, d, median [IQR]	0 [0,2.75]	0 [0,0]	0.001	0 [0,1.75]	0 [0,0]	0.012	0 [0,1.75]	0 [0,0]	0.011

Surgical outcome	TPG (AU)			PBSA (mm^2^/m^2^)		
	Sarcopenia	Non-Sarcopenia	*P* value	Sarcopenia	Non-Sarcopenia	*P* value
*Complication*						
CD grade ≥ 2, *n* (%)	33 (58.9)	56 (32.6)	< 0.001	32 (57.1)	57 (33.1)	0.001
CD Grade ≥ 3, *n* (%)	12 (21.4)	22 (12.8)	0.115	15 (26.8)	19 (11.0)	0.004
Respiratory infection, *n* (%)	20 (35.7)	30 (17.4)	0.004	21 (37.5)	29 (16.9)	0.001
Abdominal infection, *n* (%)	13 (23.2)	27 (15.7)	0.199	9 (16.1)	31 (18.0)	0.739
Wound infection, *n* (%)	5 (8.9)	8 (4.7)	0.231	5 (8.9)	8 (4.7)	0.231
Leakage, *n* (%)	4 (7.1)	8 (4.7)	0.468	4 (7.1)	8 (4.7)	0.468
Ideal Outcome, *n* (%)	37 (66.1)	155 (90.1)	< 0.001	35 (62.5)	157 (91.3)	< 0.001
Mortality at 30-days, *n* (%)	18 (32.1)	14 (8.1)	< 0.001	19 (33.9)	13 (7.6)	< 0.001
Mortality at Hospital, *n* (%)	0 (0.0)	4 (2.3)	0.250	0 (0.0)	4 (2.3)	0.250
Readmission with 30-days, *n* (%)	1 (1.8)	7 (4.1)	0.420	2 (3.6)	6 (3.5)	0.977
Length of stay, d, median [IQR]	9 [6, 15]	9 [7, 12]	0.308	9 [6, 15]	9 [7, 12]	0.994
ICU stay, d, median [IQR]	0 [0,2.75]	0 [0,0]	0.005	0 [0,3]	0 [0,0]	< 0.001

*PML3* psoas muscle to L3 vertebral body ratio, *PMI* psoas muscle index, *PMD* psoas muscle density, *TPG* total psoas gauge, *PBSA* psoas muscle to body face area ratio, *SD* standard deviation, *IQR* interquartile range. *Chi-square test* category parameters, *T test or Mann–Whitney U test* consecutive parameters, *CD grade* Clavien-Dindo grade, *ICU* intensive care unit

*P* value < 0.05 was considered statistically significant

The sarcopenia defined by each psoas major measurement value was closely related to the ideal outcome defined by us and mortality at 30-days. The same result also applied to the length of ICU stay.

### Univariate analysis

We performed a univariate regression analysis including the factors related to the prognosis (Table 6).

**Table 6:** Univariate analysis

	Ideal outcome		Mortality at 30-days		Complication (CD Grade ≥ 2)	
	OR (95% CI)	*P* value	OR (95% CI)	*P* value	OR (95% CI)	*P* value
Age	0.942 (0.915–0.971)	< 0.001	1.057 (1.025–1.090)	< 0.001	1.025 (1.007–1.044)	0.007
Gender	0.457 (0.204–1.024)	0.057	1.800 (0.792–4.093)	0.161	0.637 (0.370–1.097)	0.104
CCI ≥ 1	0.262 (0.110–0.628)	0.003	3.913 (1.542–9.926)	0.004	1.792 (1.035–3.101)	0.037
Malignancy	0.291 (0.134–0.632)	0.002	3.642 (1.631–8.130)	0.002	2.197 (1.129–4.276)	0.021
Previous abdominal surgery	0.449 (0.218–0.922)	0.029	1.952 (0.92–4.143)	0.082	1.749 (1.015–3.016)	0.044
Peritoneal soiling	0.818 (0.395–1.695)	0.589	1.303 (0.604–2.812)	0.500	1.610 (0.933–2.781)	0.087
Surgery (minor/major)	0.834 (0.408–1.706)	0.619	1.234 (0.582–2.619)	0.584	1.442 (0.844–2.465)	0.180
Sepsis	0.030 (0.007–0.129)	< 0.001	58.353 (7.796–436.784)	< 0.001	4.472 (2.533–7.895)	< 0.001
BMI	1.082 (0.973–1.203)	0.146	0.902 (0.805–1.011)	0.076	0.949 (0.879–1.025)	0.186
ASA score (≥ III/I, II)	0.232 (0.097–0.554)	0.001	5.625 (2.081–15.206)	0.001	3.471 (1.960–6.148)	< 0.001
ALB > 35 g/L	2.932 (1.409–6.101)	0.004	0.278 (0.127–0.611)	0.001	0.419 (0.242–0.726)	0.002
PML3 (low/high)	0.159 (0.075–0.340)	< 0.001	6.281 (2.848–13.852)	< 0.001	2.972 (1.598–5.528)	0.001
PMI (low/high)	0.214 (0.101–0.450)	< 0.001	4.562 (2.096–9.931)	< 0.001	2.210 (1.198–4.077)	0.011
PMD (low/high)	0.329 (0.156–0.692)	0.003	3.336 (1.537–7.240)	0.002	2.005 (1.089–3.693)	0.026
TPG (low/high)	0.214 (0.101–0.450)	< 0.001	5.346 (2.443–11.698)	< 0.001	2.972 (1.598–5.528)	0.001
PBSA (low/high)	0.159 (0.075–0.340)	< 0.001	6.281 (2.848–13.852)	< 0.001	2.690 (1.451–4.987)	0.002

*OR* odds ratio, *CI* confident interval, *CCI* Charlson Comorbidities Index, *BMI* body mass index, *ASA* American Society of Anesthesiologists, *ALB* albumin, *HB* hemoglobin. *PML3* psoas muscle to L3 vertebral body ratio, *PMI* psoas muscle index, *PMD* psoas muscle density, *TPG* total psoas gauge, *PBSA* psoas muscle to body face area ratio

*P* value < 0.05 was considered statistically significant

Each psoas muscle calculation was related to the ideal outcome, mortality at 30-days, and the occurrence of complications with a Clavien-Dindo score ≥ 2.

### Multivariate analysis

We performed a multivariate logistic regression analysis on the ideal outcome and mortality at 30-days (Table 7). The included variables comprised Age, Charlson Comorbidity Index, sepsis, and sarcopenia defined by each type of psoas calculation.

**Table 7:** Multivariate analysis: PMI versus PML3 versus PMD versus TPG versus PBSA

Ideal outcome	PML3		PMI		PMD	
	OR (95% CI)	*P* value	OR (95% CI)	*P* value	OR (95% CI)	*P* value
Age ≥ 65	0.275 (0.104–0.729)	0.009	0.194 (0.073–0.515)	0.001	0.213 (0.085–0.531)	0.001
CCI ≥ 1	0.290 (0.096–0.874)	0.028	0.308 (0.105–0.904)	0.032	0.371 (0.134–1.022)	0.055
Sepsis	0.019 (0.004–0.094)	< 0.001	0.021 (0.004–0.099)	< 0.001	0.024 (0.005–0.109)	< 0.001
Sarcopenia	0.160 (0.058–0.436)	< 0.001	0.176 (0.065–0.480)	0.001	0.514 (0.204–1.292)	0.157

Ideal outcome	TPG		PBSA	
	OR (95% CI)	*P* value	OR (95% CI)	*P* value
Age ≥ 65	0.233 (0.091–0.595)	0.002	0.182 (0.067–0.493)	0.001
CCI ≥ 1	0.339 (0.119–0.967)	0.043	0.343 (0.116–1.011)	0.052
Sepsis	0.024 (0.005–0.110)	< 0.001	0.020 (0.004–0.100)	< 0.001
Sarcopenia	0.264 (0.102–0.681)	0.006	0.159 (0.059–0.427)	< 0.001

Mortality at 30-days	PML3		PMI		PMD	
	OR (95% CI)	*P* value	OR (95% CI)	*P* value	OR (95% CI)	*P* value
Age ≥ 65	2.386 (0.886–6.422)	0.085	3.334 (1.265–8.790)	0.015	3.186 (1.263–8.038)	0.014
CCI ≥ 1	3.691 (1.180–11.547)	0.025	3.395 (1.116–10.326)	0.031	2.866 (0.996–8.247)	0.051
Sepsis	78.036 (9.667–629.942)	< 0.001	70.958 (8.964–561.702)	< 0.001	64.746 (8.396–499.279)	< 0.001
Sarcopenia	6.326 (2.287–17.499)	< 0.001	4.983 (1.842–13.477)	0.002	2.256 (0.885–5.748)	0.088

Mortality at 30-days	TPG		PBSA	
	OR (95% CI)	*P* value	OR (95% CI)	*P* value
Age ≥ 65	2.827 (1.081–7.391)	0.034	3.538 (1.317–9.509)	0.012
CCI ≥ 1	3.213 (1.069–9.662)	0.038	3.111 (1.018–9.506)	0.046
Sepsis	66.086 (8.437–517.681)	< 0.001	71.272 (8.880–572.032)	< 0.001
Sarcopenia	4.571 (1.732–12.065)	0.002	5.712 (2.133–15.295)	0.001

*PML3* psoas muscle to L3 vertebral body ratio, *PMI* psoas muscle index, *PMD* psoas muscle density, *TPG* total psoas gauge, *PBSA* psoas muscle to body face area ratio, *OR* odds ratio, *CI* confident interval, *CCI* Charlson Comorbidities Index

*P* value < 0.05 was considered statistically significant

In all regression models, only PMD (*P* value = 0.157 and 0.088, respectively) was not an independent risk factor.

**Fig. 3:**
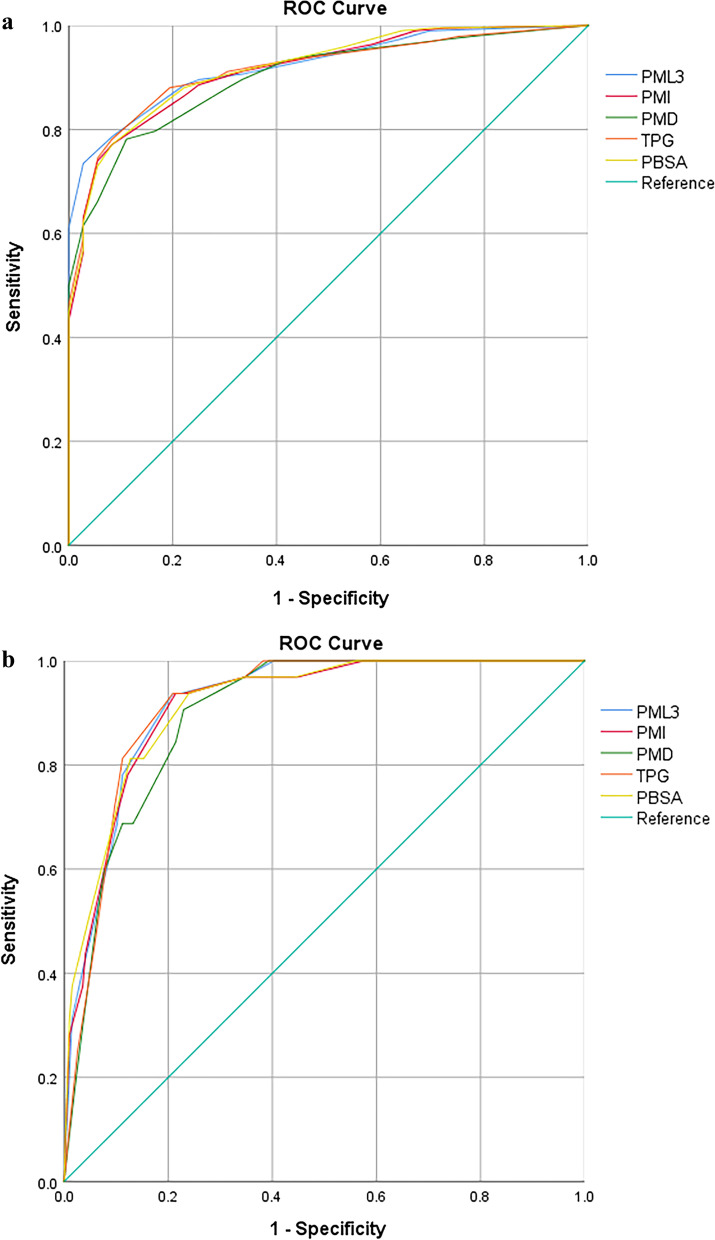
ROC analysis result. **a** ROC curves of the ideal outcome logistic models. **b** ROC curves of the mortality at 30-days logistic models

We performed ROC analysis for each model and calculated the AUC (Table 8 and Fig. 3). Among the ideal outcome prediction models, PML3 model has the largest AUC value (AUC = 0.922, 95% CI 0.886–0.958). The same result applies to the mortality at 30-days prediction model (AUC = 0.920, 95% CI 0.881–0.959). In pairwise DeLong test, no statistical significance was observed in pairwise comparison of AUC for each model (Additional file 1: Table S1).

**Table 8:** AUC value of each logistic model

	Ideal outcome	Mortality at 30-days
	AUC (95% CI)	AUC (95% CI)
PML3	0.922 (0.886–0.958)	0.920 (0.881–0.959)
PMI	0.914 (0.873–0.956)	0.915 (0.872–0.959)
PMD	0.900 (0.856–0.944)	0.899 (0.855–0.943)
TPG	0.914 (0.873–0.955)	0.917 (0.879–0.956)
PBSA	0.918 (0.877–0.959)	0.917 (0.874–0.961)

## Discussion

To our knowledge, this study is the first to evaluate the relationship between CT-defined sarcopenia and the clinical outcome of emergency laparotomy in an East Asian population. The conclusion is similar to the previous studies in European and American populations [2, 12–17]. The occurrence of sarcopenia could predict a poor outcome of emergency laparotomy.

This study is also the first to compare the ability of various previously reported CT psoas major calculations to predict the clinical outcome of emergency laparotomy. PML3 model might perform better in predicting prognosis than other models according to our results. However, no statistical significance was shown in pairwise comparisons with the models' AUC values, which indicated that they have similar performance in outcome prediction.

This study determined new sex-specific cut-off values for psoas major muscle measurements in patients undergoing emergency laparotomy, which are different from cut-off values for patients with gastric cancer of the same race [19]. We knew that malignant diseases will cause muscle atrophy [24–26], but the cut-off values we reported were not generally higher than cancer patients as expected, even lower. In our study, the included patients came from the largest medical center in central China, and most of them were critical cases with poor general status. It may be one of the reasons that can explain this phenomenon. A large sample of epidemiological studies may be needed to determine the sex-specific cut-off values for CT diagnosis of sarcopenia.

Postoperative complications are another clinical outcome of concern besides mortality. Our study combined all the patients with a bad status and defined the "ideal outcome" as our primary outcome variable. Compared to 1-dimensional postoperative outcome parameters like mortality, such a composite measure can better reflect patients' prognosis [27–29].

Sarcopenia increased the risk of postoperative infection. This conclusion has been proven in previous work [30, 31]. In our study, sarcopenia defined by each psoas muscle calculation was related to respiratory infection; but the same result cannot be applied to abdominal infection.

Obviously, the different stages of contrast-enhanced CT will affect the determination of skeletal muscle density [32]. To our knowledge, no previous studies have shown whether artificial implants have an effect on the skeletal muscle measurement determined by CT. In this study, we chose to exclude patients with artificial implants to avoid possible interference.

Hajibandeh et al. completed a meta-analysis of the impact of sarcopenia on the prognosis of emergency laparotomy. Four studies from North America and the United Kingdom were included. The results showed that sarcopenia could be an independent risk factor for poor prognosis for emergency laparotomy [33]. Contrary to most studies, Dirks et al. incorporated psoas TPA, PMI, PMD, and other parameters into the multivariate analysis and found that these measurements cannot be used as independent risk factors for mortality. It may be because they chose to collect relevant parameters at the L4 level [34] instead of the L3 level chosen by most studies. Additionally, in studies with positive results, the levels chosen were not precisely the same. In our study, due to the calculation requirements of PML3, we referred to the inferior end-plate level of the L3 vertebra selected by Simpson et al. [12, 13, 17]. There were also other studies that chose the L3 level that makes the two transverse processes of the third lumbar vertebra visible[16, 22].

In our study, sarcopenia defined by PMD cannot be used as an independent risk factor for clinical outcome in a multivariate analysis (*P* value = 0.157, 0.088, respectively). In the study of Tzeng et al., PMD can be used as an independent risk factor for the postoperative hospital stay in patients undergoing transcatheter aortic valve implantation [35]. In the study of Salem et al., PMD can also be used as an independent risk factor for emergency laparotomy [16]. The population difference might be one of the reasons to explain this. Further research may be needed to confirm the effectiveness of psoas muscle density in risk prediction.

In the practice of surgery, researchers have developed various surgical risk prediction models, such as Portsmouth Physiological and Operative Severity Score for the enUmeration of Mortality (P-POSSUM) and National Emergency Laparotomy Audit (NELA) models [3, 36]. However, the previous prognostic scoring model of emergency surgery generally lacks the inclusion of the parameter of "frailty"[3]. In the past, "frailty" or malnutrition had various evaluation methods, including questionnaires, functional tests, and so on [37, 38]. However, in the urgency of emergency surgery, patients are not allowed to accept such tests that mix subjective factors and, more importantly, may delay the treatment. Sarcopenia is related to physical frailty and can be used as an evaluation indicator of "frailty" [39, 40]. In addition, as a routine examination of patients for diagnosis before surgery, CT has unique advantages [41]. Moreover, in clinical applications, the measurement of the total cross-sectional skeletal muscle area [14] often requires professional imaging software and complex processes such as extracting Digital Imaging and Communications in Medicine (DICOM) files, while the collection of psoas muscle measurement values is more convenient and worthy of promotion in clinical work, especially in less developed countries. [42].

Models that use CT psoas muscle measurements as one of the variables will improve the capabilities of the prognostic model [12, 13, 17]. Simpson et al. tried to include PML3 in the P-POSSUM model, which improved the model's ability to predict mortality [17]. Body et al. also made a similar attempt. They included CT-defined sarcopenia and myosteatosis as variables in the NELA model, which also improved the model's predictive ability [14]. In the model we constructed, the Nagelkerke *R*^2^ values were larger in the model with the sarcopenia parameter than in the model without (Additional file 1: Table S2). The inclusion of the sarcopenia parameter generally improved the model. We would recommend adding sarcopenia as a novel parameter in the prognostic model for emergency laparotomy in the future.

## Limitation

There are some limitations in our study, such as the retrospective nature, a certain degree of data loss, relatively small population samples, and heterogeneous management methods for patients, which may affect the study results.

We did not prospectively collect the variables needed for other scoring systems (such as NELA, P-POSSUM models), so it was unlikely to evaluate whether the model's predictive ability would be improved by including the psoas muscle measurements as variables. We did not follow up with the patients for a long time, so we cannot evaluate the long-term clinical outcome in this study. We also did not prospectively collect parameters such as nutritional scores or muscle strength measurements to evaluate the patient's skeletal muscle state, so it was impossible to evaluate whether the sarcopenia determined by CT and the set cut-off values were consistent with the clinical diagnosis.

## Conclusion

The measured values of psoas major muscle determined by CT, except PMD, can be used as an independent risk factor for the prognosis of emergency laparotomy. Large sample research may be needed to accurately determine the CT psoas muscle measurement value as the cut-off value of the diagnostic criteria for sarcopenia. A prognostic model including a sarcopenia parameter should be developed in the future.

## Supplementary Information

**Additional file 1:** The statistical evaluation of the models.

## Data Availability

The study datasets are available from the corresponding author on reasonable request.

## Abbreviations

EL: Emergency laparotomy
CT: Computed tomography
PMI: Psoas muscle index, PML3: psoas muscle to L3 vertebral body ratio
PMD: Psoas muscle density
TPG: Total psoas gauge
PBSA: Psoas muscle to body face area ratio
CCI: Charlson Comorbidities Index
SD: Standard deviation
IQR: Interquartile range
ROC: Receiver operating characteristics
AUC: Area under curve
EWGSOP: European Working Group on Sarcopenia in Older People
MRI: Magnetic resonance imaging
BMI: Body mass index
ASA: American Society of Anesthesiologists
ROI: Area of interest
TPA: Total psoas area
OR: Odds ratio
NELA: National Emergency Laparotomy Audit
P-POSSUM: Portsmouth Physiological and Operative Severity Score for the enUmeration of Mortality
DICOM: Digital Imaging and Communications in Medicine
