# Supplementary material for: CT psoas calculations on the prognosis prediction of emergency laparotomy: a single-center, retrospective cohort study in eastern Asian population
Source: World J Emerg Surg. 2022 Jun 3;17:31. doi: 10.1186/s13017-022-00435-x (PMC9164461; doi:10.1186/s13017-022-00435-x)
Supplement: Supplementary file 1 — Additional file 1: The statistical evaluation of the models. [file 13017_2022_435_MOESM1_ESM.docx]

**Additional file 1**

**Table S1.** Pairwise DeLong test of models' AUC values

|  |  | **Ideal outcome** | |  | **Mortality at 30-days** | |
| --- | --- | --- | --- | --- | --- | --- |
|  |  | AUC difference value(95% C.I.) | Sig. |  | AUC difference value(95% C.I.) | Sig. |
| PML3-PMI |  | 0.008(-0.013-0.029) | 0.466 |  | 0.005(-0.013-0.023) | 0.590 |
| PML3-PMD |  | 0.022(-0.004-0.048) | 0.096 |  | 0.021(-0.005-0.047) | 0.112 |
| PML3-TPG |  | 0.008(-0.012-0.028) | 0.434 |  | 0.003(-0.016-0.022) | 0.786 |
| PML3-PBSA |  | 0.004(-0.018-0.026) | 0.712 |  | 0.003(-0.017-0.022) | 0.798 |
| PMI-PMD |  | 0.014(-0.013-0.042) | 0.303 |  | 0.016(-0.012-0.044) | 0.268 |
| PMI-TPG |  | 0.000(-0.026-0.026) | 0.983 |  | -0.002(-0.024-0.020) | 0.835 |
| PMI-PBSA |  | -0.004(-0.018-0.011) | 0.628 |  | -0.002(-0.019-0.014) | 0.772 |
| PMD-TPG |  | -0.014(-0.032-0.004) | 0.117 |  | -0.018(-0.042-0.006) | 0.133 |
| PMD-PBSA |  | -0.018(-0.045-0.009) | 0.191 |  | -0.018(-0.046-0.009) | 0.190 |
| TPG-PBSA |  | -0.004(-0.030-0.022) | 0.768 |  | 0.000(-0.021-0.021) | 0.994 |

**Table S2.** Nagelkerke R² and Hosmer Lemeshow test results of each model (Adding sarcopenia parameters to the models itself generally improves according to the Nagelkerke R² results)

|  | **Ideal outcome** | |  | **Mortality at 30-day** | |
| --- | --- | --- | --- | --- | --- |
|  | Nagelkerke R² | Hosmer Lemeshow Test |  | Nagelkerke R² | Hosmer Lemeshow Test |
| PML3 | 0.545 | 0.826 |  | 0.529 | 0.933 |
| PMI | 0.537 | 0.987 |  | 0.512 | 0.914 |
| PMD | 0.482 | 0.985 |  | 0.468 | 0.911 |
| TPG | 0.513 | 0.916 |  | 0.507 | 0.714 |
| PBSA | 0.548 | 0.997 |  | 0.525 | 0.803 |
| Without sarcopenia parameter | 0.471 | 0.979 |  | 0.451 | 0.978 |
